# Retzius-sparing robot-assisted radical prostatectomy is safe for patients with prior transurethral prostate surgery

**DOI:** 10.1590/S1677-5538.IBJU.2017.0316

**Published:** 2018

**Authors:** Lawrence H. C. Kim, Glen Denmer Santok, Ali Abdel Raheem, Kidon Chang, Trenton Lum, Byung Ha Chung, Young Deuk Choi, Koon Ho Rha

**Affiliations:** 1 Department of Urology and Urological Science Institute, Yonsei University College of Medicine, Seoul, South Korea; 2 Department of Urology, Tanta University Medical School, Egypt

## INTRODUCTION

Several studies have shown that patients with prior transurethral prostate surgery are associated with greater perioperative complications, as well as inferior oncological and functional outcomes when they undergo robot-assisted radical prostatectomy (RARP).

Objectives: To the best of our knowledge, there is no study thus far evaluating the association between prior transurethral prostate surgery and the above outcomes following Retzius-sparing robot-assisted radical prostatectomy (RS-RARP).

## MATERIALS AND METHODS

A retrospective review of 413 patients who underwent RS-RARP by a single surgeon from November 2012 to December 2015 was analyzed. There are no certain selection criterions to perform or not Retzius-sparing approach. Patients were divided into two groups based on the history of prior transurethral surgery. Patient clinicopathological characteristics, perioperative outcomes as well as short term oncological outcome and continence rates up to one year post RS-RARP were compared between the two groups.

## RESULTS

Seventeen patients (4.1%) underwent prior transurethral prostate surgery. There was no difference in the baseline patient clinicopathological characteristics apart from older age in the TURP group. Perioperative, and oncological outcomes were comparable between the groups, Continence rates at one month, three months, six months and one year post RS- RARP were also similar between the two groups.

## CONCLUSION

Equivalent perioperative, oncological and functional outcomes were achieved between the two groups. RS RARP is a safe and feasible option following previous transurethral prostate surgery.

## ARTICLE INFO

**Available at: http://www.intbrazjurol.com.br/video-section/20170316_Kim_et_al**

**Int Braz J Urol. 2018; 44 (Video #11): 842-3**

## Video abbreviations

B: Bladder
P: Prostate
U: Urethra
BN: Bladder neck
DF: Denoviller's fascia
PF: Pelvic Floor
NVB: Neurovascular bundle
DA: detrusor apron

